# Temperature-Dependent Trimethylamine *N*-Oxide Induced the Formation of Substance P Dimers

**DOI:** 10.1021/acs.jpcb.4c04951

**Published:** 2024-11-06

**Authors:** Carter Lantz, Zhenyu Xi, Robert L. Rider, Thomas E. Walker, Michael Hebert, David H. Russell

**Affiliations:** Department of Chemistry, Texas A&M University, College Station, Texas 77843, United States

## Abstract

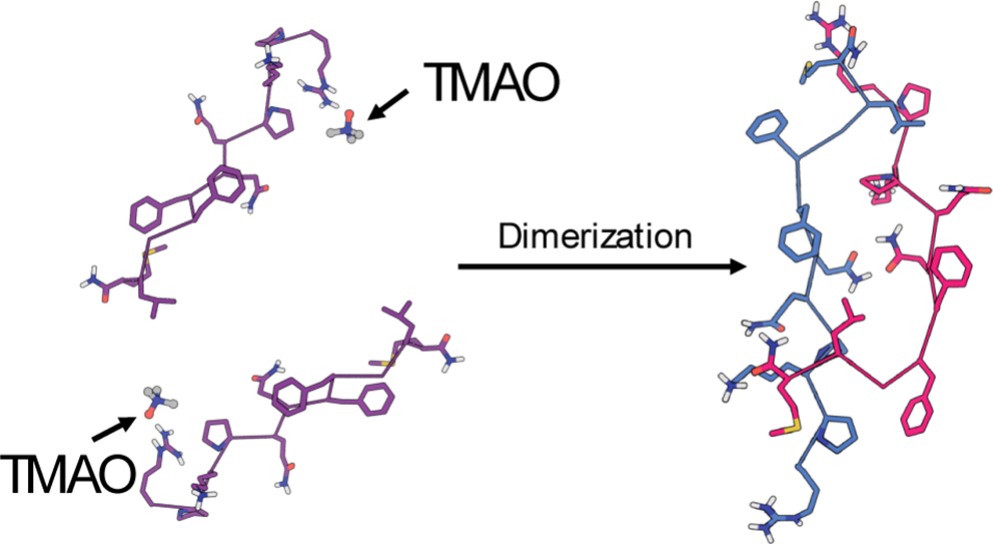

Interactions of the peptide substance P (SP) (RPKPQQFFGLM-NH_2_) with trimethylamine *N*-oxide (TMAO) were investigated by using cryo-ion mobility-mass spectrometry (cryo-IM-MS), variable-temperature (278–358 K) electrospray ionization (vT-ESI) MS, and molecular dynamics (MD) simulations. Cryo-IM-MS provides evidence that cold solutions containing SP and TMAO yield abundant hydrated SP dimer ions, but dimer formation is inhibited in solutions that also contain urea. In addition, we show that SP dimer formation at cold solution temperatures (<298 K) is favored when TMAO interacts with the hydrophobic C-terminus of SP and is subject to reduced entropic penalty when compared to warmer solution conditions (>298 K). MD simulations show that TMAO lowers the free energy barrier for dimerization and that monomers dimerize by forming hydrogen bonds (HBs). Moreover, differences in oligomer abundances for SP mutants (P4A, P2,4A, G9P, and P2,4A/G9P) provide evidence that oligomerization facilitated by TMAO is sensitive to the *cis*/*trans* orientation of residues at positions 2, 4, and 9.

## Introduction

Structures of peptides and proteins are dictated by interactions with cofactors, osmolytes, and other peptides and proteins and intramolecular interactions involving hydrophobic and hydrophilic interactions that are often dictated by interactions with solvent. Dissecting contributions for each of these interactions and determining how their collective effects alter protein dynamics and conformational preferences represent major challenges for biophysics.^1−3^ Here, we report results from experiments aimed at delineating the effects of osmolytes (TMAO and urea) that have been shown to influence stabilities, dynamics, and conformational entropy of peptides and proteins, viz., the balance between folded and unfolded states.^4−6^ The general view is that TMAO influences structure by altering hydration of the protein, thereby entropically favoring folded conformations through hydrophobic interactions,^6,7^ and these effects have been shown to counteract the denaturing effects of urea.^6,8^ While much has been learned from MD simulations concerning the effects of osmolytes on protein structure, stabilities, and dynamics, experimental data to support specific mechanisms, viz., mechanisms that involve direct or indirect peptide–osmolyte interactions, are limited. Direct interactions are thought to disrupt the local structure of proteins through interactions with the amino acid side chains or the peptide backbone, whereas indirect mechanisms involve reorganization of the surrounding solvent.^9−11^

Investigations delineating how TMAO and temperature influence conformational and compositional preferences were motivated by our prior studies that illustrated the effects of hydration on the conformational preferences of the undecapeptide SP (RPKPQQFFGLM–NH_2_).^12,13^ Results from these studies provided evidence that [SP + 3H]^3+^ ions formed by electrospray ionization (ESI) reveal a remarkable degree of conformational heterogeneity. At least two conformational states were identified: (i) compact conformers that are formed by evaporation of water from hydrated ions and (ii) elongated conformers that are formed by unfolding of the dehydrated compact conformers.^12^ MD simulations further revealed that these compact conformers are stabilized by intramolecular interactions involving charge-carrying sites, viz., the arginine (R^1^) and lysine (K^3^) side chains, polar glutamine (Q^5^Q^6^) side chains, and nonpolar phenylalanine (F^6^F^7^) side chains.^12^ Previous work also provided evidence that conformational preferences of [SP + 3H]^3+^ ions are dependent on whether the configuration of prolines (P^2^ and P^4^) is *cis* or *trans*; P → A mutations (P^2^/A, P^4^/A) and the double mutant P^2^, P^4^/A produced significantly different collision cross section (CCS) values from that of the native [SP + 3H]^3+^ ions.^12^ The addition of a proline at position 9 also shifted the conformer distribution of the peptide due to the change in configuration from *trans* to *cis*.^12^

**Figure 1:**
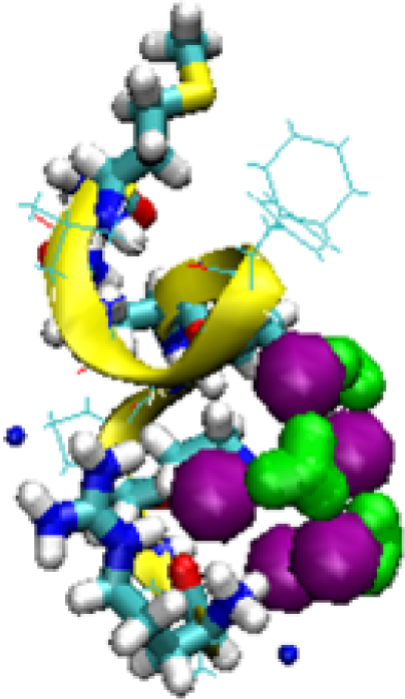
Structure from an MD simulation showing the structure of SP and the accumulation of H_3_O^+^ and Cl^–^ ions (green and purple regions correspond to H_3_O^+^ and Cl^–^ ions bound to the *N*-terminus, respectively).^13^

MD simulations were also used to model changes in the conformational preferences of the [SP + 3H]^3+^ ions during the transition from a hydrated environment to the solvent-free [SP + 3H]^3+^ ions.^13^ Here, we tracked the transition of a nanodroplet composed of ∼2400 water molecules containing 22 hydronium ions (H_3_O^+^), 10 chloride ions (Cl^–^), and a single [SP + 3H]^3+^ ion to the final stages of dehydration.^13^ In the early stage of droplet shrinkage, a combination of solvent evaporation and ejection of excess charge, primarily hydrated H_3_O^+^and Cl^–^, were observed, which were attributed to droplet charge-induced fission events.^13^ [SP + 3H]^3+^ ions adapt to changes in the droplet size through small conformational changes that result in coiling of the hydrophobic C-terminus of the peptide at or near the droplet surface. The hydrated hydrophilic N-terminus forms compact structures involving intramolecular interactions and water-mediated interactions between the [SP + 3H]^3+^ charge-carrying sites and H_3_O^+^ and Cl^–^ (Figure 1). CCS measurements calculated by MD simulations for [SP + 3H]^3+^ ions at various stages of desolvation are consistent with measurements from cryogenic ion mobility-mass spectrometry (cryo-IM-MS).^13^ Specifically, [SP + 3H]^3+^ ions favor an extended conformation in the early stages of droplet decay, whereas the ions favor a more compact conformation in the final stages of dehydration. Here, we extend these studies on the effects of osmolytes (TMAO and urea) on the dimerization of SP using cryo-IM-MS, vT-ESI, and MD simulations, and our results provide evidence that osmolytes shift the hydration shell around SP ions, which alters the amount of dimer formation.

## Materials and Methods

### Substance P Oligomerization Experiments

SP and SP mutants were obtained from Sigma-Aldrich (St. Louis, MO) at a purity of 95%, from American Peptide (Sunnyvale, CA), or from Mocell (Shanghai, China). The TMAO used in this study was obtained from Cayman Chemical (Ann Arbor, MI) at a purity of 95%. The urea used in this study was purchased from EM Science at a purity of 99%. Unless otherwise noted, all solutions used in this work were prepared in 18.2 MΩ water at pH ∼ 7.

All vT-ESI experiments were conducted on a Waters Synapt-G2 TWIMS-ToF instrument (Milford, MA). We previously described methodologies for using vT-ESI-MS experiments to measure the thermodynamics of proteins and their proteoforms.^14,15^ The concentration of SP was maintained at 5 μM, while the TMAO concentration was varied. Typical SP to TMAO ratios were 50:1, 5:1, 1:1, 1:10, and 1:100. Values for the thermodynamic terms for the formation of the [2SP + 2H]^2+^ dimer and [SP + 2H]^2+^/TMAO complex were calculated using the relative abundance of the precursors and product ions as determined from the IM-MS spectra.^16−18^ The equilibrium constants for the SP/TMAO interaction were calculated in a similar manner. Plots of the natural log of the equilibrium constants, ln(*K*_a_), versus the inverse of the solution temperature yield the van’t Hoff relationship, from which the Δ*G*, Δ*H,* and −TΔ*S* values were calculated. The thermodynamic study was conducted in triplicate, and all error bars are representative of the standard deviation of the measurements.

SP mutant oligomer experiments were performed by dissolving 50 μM SP in water with and without 1 mM TMAO. Mass spectra of the solutions were collected on an Exactive Plus EMR, Thermo Fisher Scientific (Waltham, MA). Oligomer analysis was completed using Protein Metrics (Cupertino, CA) “intact” software to quantify the oligomer abundances up to the trimer due to limitations of the software to analyze higher order oligomers.

### Explanation of Thermodynamics Quantitation

Van’t Hoff plots yield thermodynamic information by plotting the natural log of the equilibrium constant (association constant) of the reaction against the inverse of the temperature in kelvin. When the points are fitted linearly, the equation of the line is in the form of eq 1.
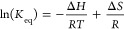
1

The equilibrium constants used to form the van’t Hoff plots were the association constants (*K*_a_). The calculation of the *K*_a_ values for the dimer formation utilized eq 2,
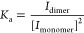
2where *I*_dimer_ is the intensity of the dimer in the mobility spectrum and *I*_monomer_ is the intensity of SP monomer in the mobility spectrum. The monomer value is squared because the “free ligand” concentration that would typically be a variable in the equation is the SP monomer itself.

Equation 3 was used to calculate the *K*_a_ values for the SP and TMAO interaction:
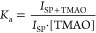
3where *I*_SP+TMAO_ is the mass spectral intensity of [SP + TMAO]^2+^, *I*_SP_ is the mass spectral intensity of the SP monomer ions, and [TMAO] is the concentration of TMAO in the sample.

### Molecular Dynamics Simulations

Simulations were conducted using the GROMACS 2023.2 program^19^ with the CHARMM36 force field,^20^ focusing on the [SP + 2H]^2+^ ion. In this setup, the positive charge was assigned to the Arg and Lys residues, while the N-terminus was kept neutral according to the p*K*_a_ values ranking to match the charge present in the mass spectra.^21^ The neutral N-terminus and the amidated C-terminus were parametrized using CHARMM-GUI.^22^ The TMAO force field parameters were obtained from Ganguly et al.^23^ Initially, two [SP + 2H]^2+^ monomers were placed randomly within a cubic box, ensuring a minimum distance of 4 nm between them. To accommodate monomeric conformational changes and facilitate dimer formation/dissociation, the box’s dimension was set to 8 nm, which corresponded to a concentration of 6.51 mM SP. The periodic boundary condition was also applied to the box. In simulations involving TMAO, 100 TMAO molecules were positioned randomly in the box. The box was filled with TIP3P water^24^ molecules and supplemented with chloride ions to serve as counterions. Both setups, with and without TMAO, underwent an initial 100 ps NVT simulation to achieve equilibrium at 300 K, employing the V-rescale thermostat.^25^ Temperature-dependent simulations were carried out by linearly increasing/decreasing the temperature to 360 and 280 K, respectively, in NVT equilibrium. Subsequently, a 100 ps NPT simulation was performed, and the Parrinello–Rahman barostat^26^ was utilized to maintain the pressure at 1 bar. All MD simulations were extended to 2000 ns to ensure relatively comprehensive sampling. Free energy profiles were derived from Max–Boltzmann distributions (eq 4):
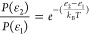
4where ε_*i*_ is the energy and *P*(ε_*i*_) is the corresponding probability of certain species. , where *N*_i_ is the number of particular frames in the simulation, is calculated based on the certain collective property (e.g., for two peptides with a certain distance, the probability would be 0.25 if this scenario were 500 ns total out of 2000 ns). Variance was estimated by bootstrapping. The center-of-mass distance was chosen as the collective property to evaluate the energy is due to its ability to distinctly differentiate dimer, monomer, and transition structures. In the FEP plot, the intrinsic coordinate is the normalized COM distance.

## Results

### TMAO Promotes Dimer Formation of Substance P

To determine how osmolytes influence dimer formation of SP, SP was added to solutions containing urea, TMAO, and TMAO with urea, and cryo-ion mobiltiy-mass spectrometry (cryo-IM-MS) was performed on the solutions. In these experiments, hydrated peptide ions formed by ESI are kinetically trapped and then analyzed by cold (80 K) IMS followed by *m*/*z* analysis by time-of-flight (ToF) MS (Figure S1).^10,11^ The abundances of SP ions ([SP + 2H]^2+^ and [SP + 3H]^3+^) produced from solutions with and without urea (Figure S2a,b) are quite similar, and there are no signals corresponding to oligomers of SP. We find no evidence that SP spontaneously forms oligomers in solution or during the ESI process in the presence or absence of urea. Cryo-IM-MS spectra for SP solutions containing a low concentration of TMAO (1 μM) contain signals for [SP + 3H]^3+^, [SP + 2H]^2+^, and SP dimer ions ([2SP + 4H]^4+^) (Figure S2c). We interpret the presence of dimers in the solution containing TMAO as evidence that TMAO promotes dimer formation of SP. Note that the abundances of both [SP + 3H]^3+^ and [2SP + 4H]^4+^ ions are reduced in solutions containing 50 μM SP, 1 μM TMAO, and 1 μM urea (Figure S2d), and a corresponding increase in the abundance of [SP + 2H]^2+^ ions is observed. The increased abundance for [SP + 2H]^2+^ ions provides evidence that in solutions containing urea, SP dimer ions are disassembled, and SP monomer ions are charge-reduced. Gault et al.^27^ previously showed that TMAO is an effective charge-reducing osmolyte, and similar results were observed by Lyu et al.,^28^ which apparently contributes to the reduced abundance of the [SP + 3H]^3+^ ions.

Results from earlier cryo-IM-MS and MD simulations show that the hydrophilic N-terminus (RPKPQQ−) of the [SP + 3H]^3+^(H_2_O)_*n*_ is hydrated (*n* = 0 to <100) and the amidated hydrophobic C-terminus (−FFGLM–NH_2_) is dry;^13^ the charge-carrying sites (peptide N-terminus and side chains of arginine (R) and lysine (K)) are stabilized by interactions with glutamine side chains (Q^5^Q^6^), and these interactions are influenced by *cis*/*trans* orientations of prolines at positions 2 and 4.^12^ The results from these prior studies provide evidence that hydration and charge sites have a significant effect on SP conformational dynamics, and the cryo-IMS studies in Figure S2 provide evidence that dimerization of SP is induced indirectly by TMAO and is not a byproduct of the ESI process; however, they also raise an important question – by what mechanism(s) do TMAO and urea alter the conformation(s), stabilities, and hydration of SP? Answers to this question cannot be obtained from cryo-IM-MS because signal-to-noise ratios (S/N) for hydrated ions are low, and hydrated ion signals are dispersed over many different signal channels. To further address this question, experiments were performed using ambient IM-MS, viz., a Waters Synapt-G2 TWIMS (traveling wave-IMS)-ToF (time-of-flight) instrument. While ambient temperature operation of this instrument does not afford direct detection of the hydrated ions, it does afford greater sensitivity and dynamic range, as well as higher mass resolution.

### Thermodynamics of SP Dimerization and TMAO Interaction

**Figure 2:**
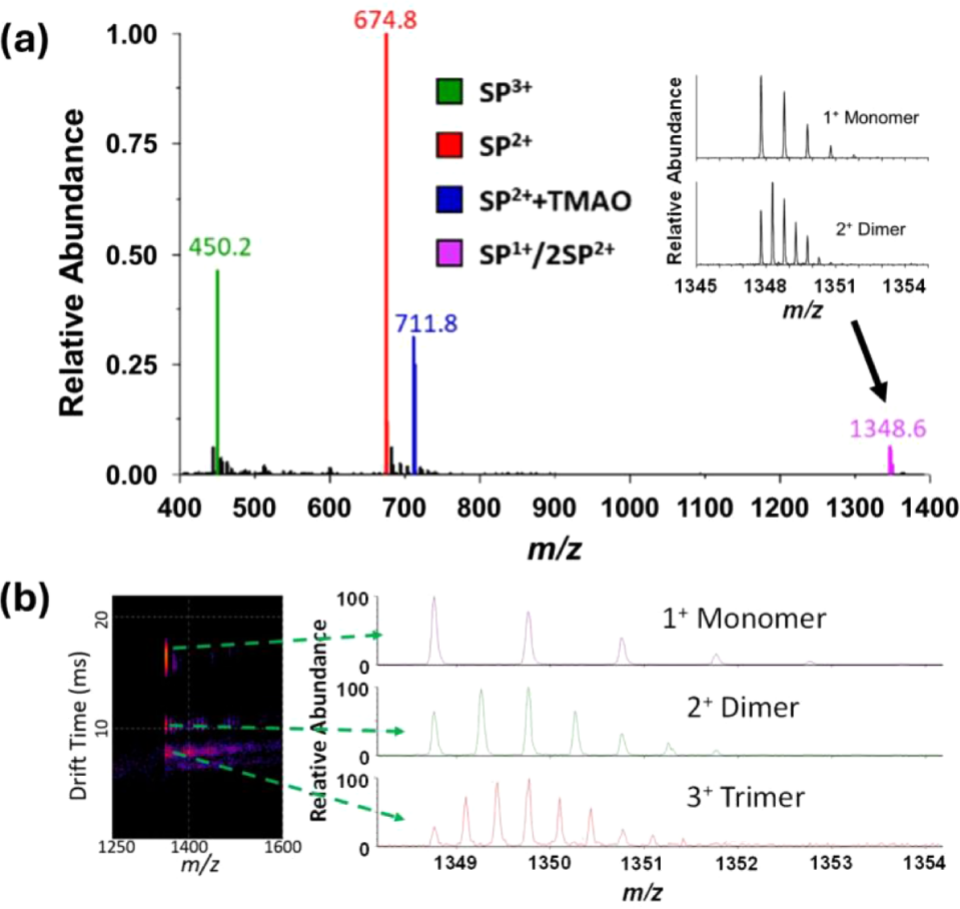
(a) A mass spectrum obtained for an aqueous solution containing 5 μM SP and 500 μM TMAO and a solution temperature of 293 K. Signals used for calculation of the thermodynamics constants are color-coded, and *m*/*z* values of the respective peaks are given above each respective peak. (b) A *m*/*z* versus drift time plot revealing multiple signals corresponding to monomers, dimers, and trimers of SP.

The Waters Synapt-G2 instrument is also equipped with variable-temperature electrospray ionization (vT-ESI) (278–358 K),^16^ which provides precise control (±2 °C) of the temperature of the solution contained in the ESI emitter. Using this approach, we have found strong temperature dependence for the formation of the [2SP + 2H]^2+^ dimer and an [SP + 2H]^2+^/TMAO complex, especially at cold temperatures (<298 K), and both reactions exhibit significant enthalpy–entropy compensation (EEC).^29,30^ Comparable thermodynamic quantities for reactions involving urea cannot be determined because these products are not observed under ambient conditions. Figure 2a contains an extracted mass spectrum obtained from a solution at 293 K containing SP and TMAO at 5 and 500 μM, respectively. [SP + H]^1+^ ions are observed when using the Synapt-G2 due to higher sensitivity compared to cryo-IM-MS and the capacity for TMAO to be an effective charge-reducing reagent.^27,28^Figure 2b contains signals for monomers, dimers, and trimers that have been extracted from the mobiligram, which provide evidence that the signals for dimer ions can be separated from those of monomers and higher-order oligomers. Increasing the concentration of TMAO in the solution led concomitantly to an increase in the relative abundances of the [SP + H]^1+^ and [2SP + 2H]^2+^ ions. Even at the lowest concentrations of added TMAO (0.1 μM/50:1 SP to TMAO ratio), product ions corresponding to SP dimers are observed. Only the 2+ dimer was observed in the mass spectra and this was thus the only charge state of the species utilized for the calculations of thermodynamic values. In neither the cryo-IM-MS nor the ambient IM-MS spectra do we find products indicative of TMAO bound to the dimer state of SP, which we interpret as evidence that TMAO does not contribute to SP dimer formation directly but rather restructures the aqueous environment around SP molecules to favor dimer formation.

**Figure 3:**
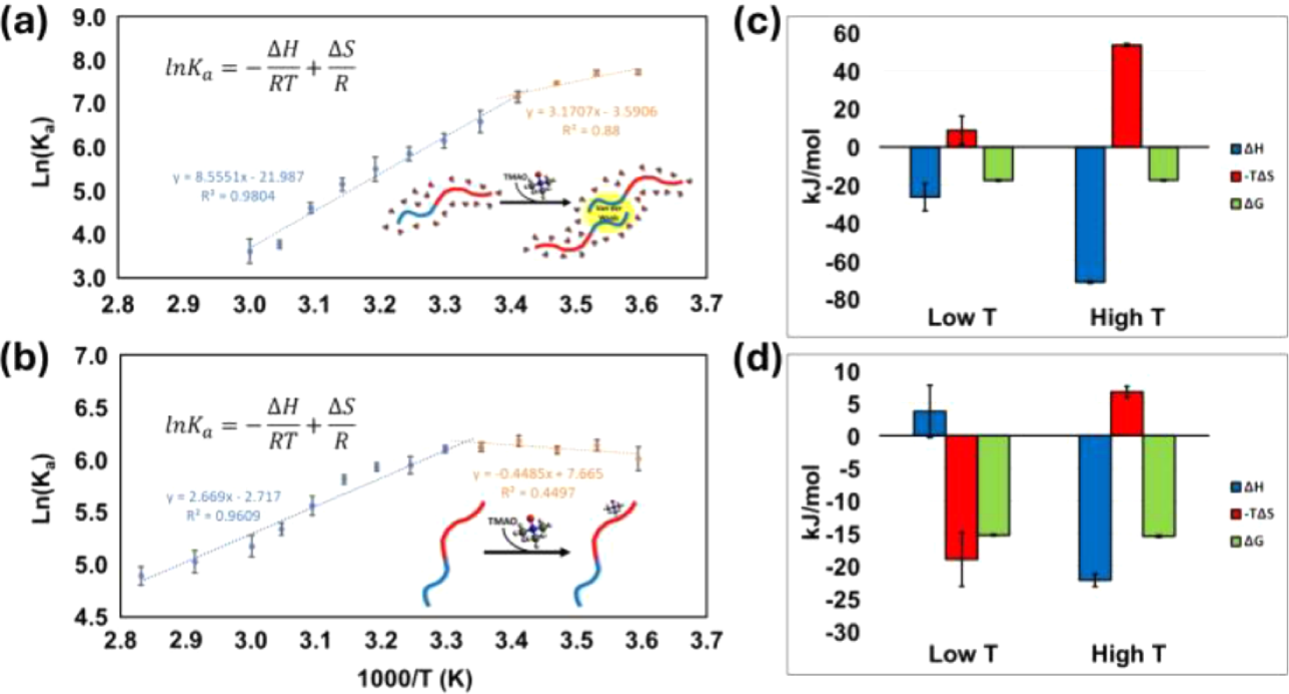
Van’t Hoff plots for (a) the self-assembly reaction of SP, and (b) the formation of the SP/TMAO complex in a solution containing SP (5 μM) and 500 μM TMAO. In both (a) and (b) the low temperature data are shown in orange and higher temperature data are shown in blue. The thermodynamic contributions of Δ*H*, -TΔ*S*, and Δ*G* for the self-assembly reaction at 293 K (c) and the formation of the SP/TMAO complex at 298 K (d) are shown. Note that the *y*-axes for (c) and (d) are not matched to scale.

Temperature dependence for SP dimer formation was investigated further using the vT-ESI-IM-MS, as described previously.^16,31^ The effects of temperature for a solution of 5 μM SP and 500 μM TMAO were analyzed using a Synapt-G2 instrument at solution temperatures between 278 to 358 K. The 278 K solution coincided with the highest relative abundance of SP dimer ions, and a further increase in the solution temperature decreased the relative abundance of dimer ions. The lowest relative abundance of dimer ions was measured at 358 K. A spectrum obtained at 293 K is shown in Figure 2a. The data suggest that lower solution temperatures favor SP self-assembly in the presence of TMAO. The discontinuity of slopes for the van’t Hoff plots (Figure 3a) is evidence that formation of SP dimers in solutions containing TMAO proceeds by different temperature-dependent mechanisms that exhibit very different EEC (Figure 3b). At temperatures greater than 293 K, favorable enthalpy overcomes the entropic penalty, resulting in an overall negative Δ*G* of −17.5 kJ/mol, and Δ*H* = −71.1 kJ/mol and −*T*Δ*S* = +53.6 kJ/mol (293 K) (Table S1). The changes in slope at temperatures less than 293 K are accompanied by very different EEC; Δ*H* and −TΔ*S* are −26.4 and +8.8 kJ/mol, respectively; and Δ*G* = −17.5 kJ/mol (Table S1). We interpret such large changes in EEC as indicators of conformational changes of the peptide and/or reorganization of the hydration of the peptide, but most likely a combination of both. At high concentrations of TMAO (500 μM), ions corresponding to the [SP + 2H]^2+^/TMAO complex (*m*/*z* 711.8) are detected (Figure 2), and the van’t Hoff plots for this product are similar to bimodal van’t Hoff plots (Figure 3b) observed for the [2SP + 2H]^2+^ complex. At low temperatures, the slope of van’t Hoff plots is negative but switches to positive at *T* > 298 K. From the fitted data, we obtain thermodynamic quantities (Δ*H* and −*T*Δ*S*) of +3.7 and −19.0 kJ/mol (298 K) for the low temperature and −22.2 and +6.5 kJ/mol (298 K), whereas Δ*G* is relatively constant, +15.3 to +15.5 kJ/mol (Table S1). Hereto, the observed EEC is indicative of temperature-dependent conformation changes; however, the effects of entropy on TMAO binding are reversed at low temperature compared to high temperature.

Van’t Hoff plots for SP dimer formation are similar, albeit less pronounced than those of [SP + 2H]^2+^/TMAO complex ions. The relative abundances of SP dimers exhibit minor changes at solution temperatures between 278 and 293 K with an inflection point at ∼293 K. The red and blue regions of the SP dimer shown in Figure 3a denote the hydrophilic (RPKPQQ−) and hydrophobic (FFGLM–NH_2_) regions of the SP amino acid sequence, respectively. The blue regions in the yellow circle denote hydrophobic interactions (“dry”) that form the SP–SP dimer (vide infra), whereas the red regions correspond to hydrated (“wet”) regions of the peptide backbone. At *T* < 293 K, formation of the SP dimer is more entropically favored, but at *T* > 293 K EEC, the entropic barrier to self-assembly increases and the process mostly becomes an enthalpically favored process as is evident by the change in slope of the van’t Hoff plot. At higher temperatures, the interactions between SP and TMAO are less favorable, as evidenced by the rapid decrease in the abundances of the [SP + 2H]^2+^/TMAO complex, an expected behavior for a reaction that is driven by favorable enthalpy. Enthalpic stabilization of proteins occurs when flexibility of hydration water is increased and the strength of HBs decreases.^32^ Thus, van der Waals interactions may contribute to the formation of the dimer at higher temperatures when the intercalation of water interrupting does not disrupt the binding region.

Formation of [SP + 2H]^2+^/TMAO ions at low temperatures is attributed to favorable hydrophobic interactions between the methyl groups of TMAO and the hydrophobic −FFGLM–NH_2_ C-terminus of SP that drives reorganization of the hydration shell of the peptide backbone, an excluded volume effect.^33^ However, at solution temperatures greater than 298 K, the formation of the [SP + 2H]^2+^/TMAO complex becomes enthalpically favored (Figure 3b). It is possible that the abrupt change in thermodynamic quantities for the formation of the [SP + 2H]^2+^/TMAO complex may correspond to a change in binding preference for the TMAO molecule, i.e., switching from limited hydrophobic binding at cold temperatures to H-bonding interactions at warmer temperatures. TMAO is readily excluded from protein surfaces, as it tends to form more favorable H-bonds with solvent.^5,34^ While the location of the TMAO adduction site(s) cannot be deduced directly from the MS data, previous MD simulations for SP confined in nanodroplets containing H_3_O^+^ and Cl^–^ anions showed clear evidence for interactions of both Cl^–^ and H_3_O^+^ ions with the charge carrying N-terminus, e.g., amino acid side chains (H_3_N^+^–RPKPQQ−);^13^ however, the experimental data indicate that Cl^–^ and H_3_O^+^ ions bind weakly to the peptide, which suggests the interaction of TMAO with hydrophilic side chains of the peptide is also weak.

### MD Simulations of SP Dimerization

**Figure 4:**
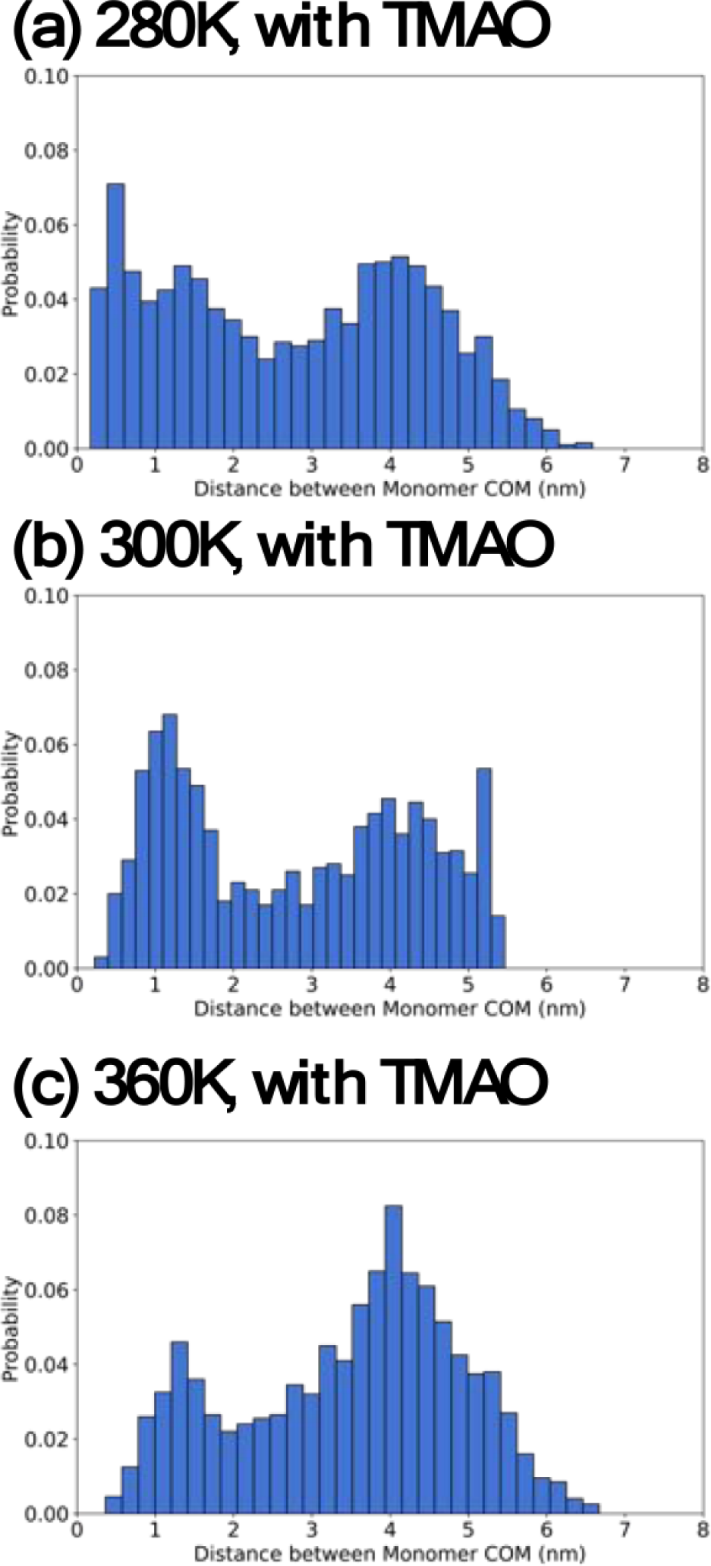
SP COM distributions at (a) 280, (b) 300, and (c) 360 K.

To further probe the effect of temperature on SP dimerization, MD simulations were performed on two SP monomer ions in a defined box with TMAO present at various temperature values, and their center of mass (COM) was measured for over 2000 ns. We determined that the SP dimer is formed when the COM distance is lower than 2 nm, and the SP monomers are fully separated when the COM distance is larger than 3.5 nm. The probability of dimer formation was 39.4% at 280 K and 39.9% at 300 K, whereas the appearance of monomers was 39.9% of the time at 280 K and 40.9% of the time at 300 K (Figure 4a,b). We interpret these results as evidence that an increase in temperature from 280 to 300 K does not affect dimer formation in the MD simulations. When the temperature was raised to 360 K, monomers were present most of the time (56.8%), while the dimer only existed for 19.7% of the total time (Figure 4c). This set of temperature-variant simulations agrees with our experimental observations; namely, the presence of the dimer was diminished at higher temperatures (Figure 3a). The prevalence of monomer species compared to dimer species at higher temperatures provides evidence that polar interactions between SP monomers could be a factor for dimer formation.

**Figure 5:**
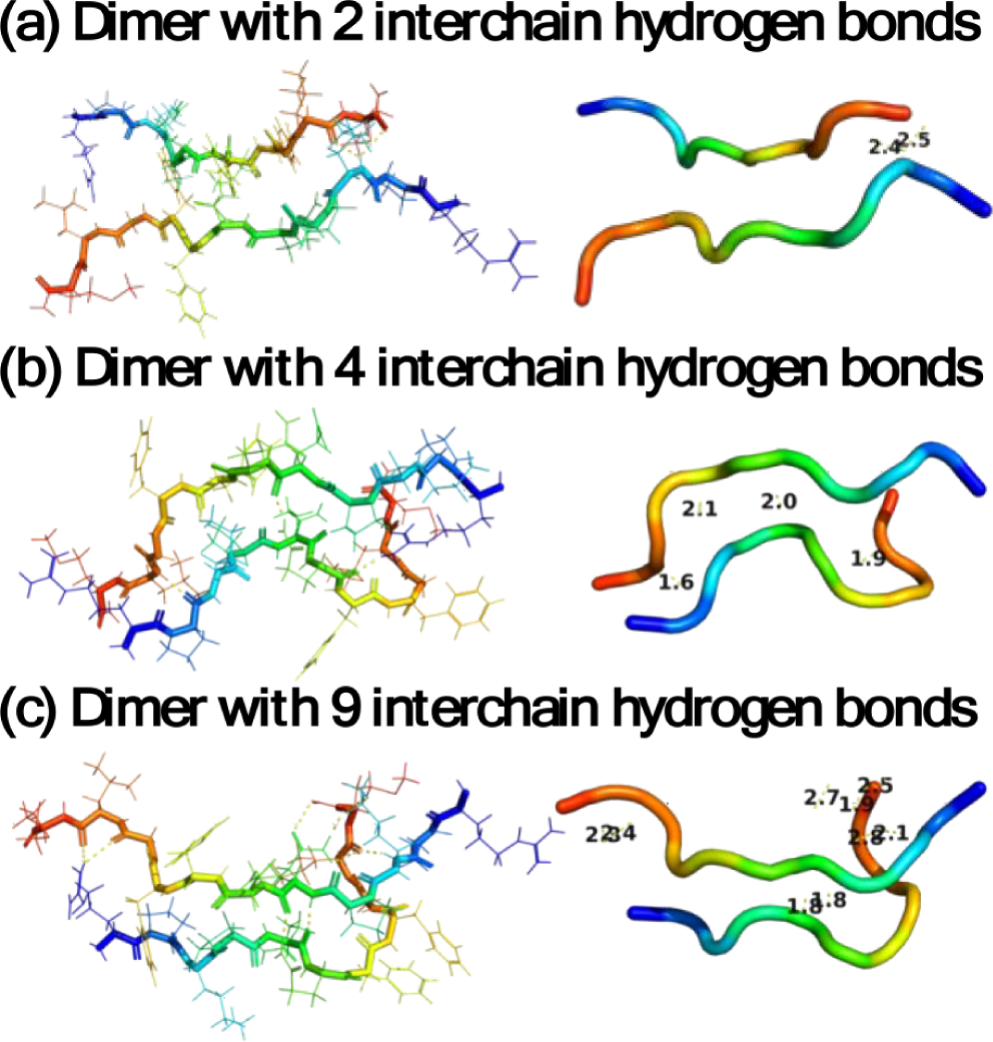
Representative antiparallel dimer formation with the aid of HBs. Each chain is colored from blue to red as moving from the N-terminus to C-terminus, where (a) 2 HB formed; (b) 4 HB formed; and (c) 9 HB formed. The numbers correspond to the distance of each hydrogen bond present between the peptides.

Further investigation of SP dimer interactions and SP/TMAO interactions with hydrogen bond (HB) analysis provides evidence that HBs play an important role in the SP interchain interaction. HB counts increase and decrease as the dimer forms and dissociates, respectively. At 300 K, a typical antiparallel conformation is formed, as the interchain HB network is established stepwise. In this scenario, the dimerization process begins with the amidated C-terminus, forming HBs with the backbone of the other monomer (Figure 5a). Subsequently, these C-terminal HBs break, but new HBs form between the side chains of Gln, Arg, and Lys and the backbone of the other monomer (Figure 5b). The structure then reorganizes to adopt a variety of HBs, including side chain–side chain, side chain–backbone, and backbone–backbone interactions (Figure 5c). HB analysis provides evidence that the dimerization process of SP is highly dynamic and involves constant formation and breaking of HBs (see SI Figure S3), which supports our conclusion in Figure 3a that attributes the formation of the dimer to enthalpic factors. Our results also provide evidence that different temperatures do not significantly affect water’s interaction with SP molecules (Figure S4). Likewise, the interaction between TMAO and SP was not significantly affected by the temperature (Figure S5).

Interactions between SP and TMAO were determined with radial distribution function (RDF) calculations, choosing hydrogens from the SP backbone, the N-terminus, the amidated C-terminus, and residues of interest (R, K, Q, F, M, and L) as reference points. The results provide evidence that (1) TMAO has a chance to form H bonds with backbone hydrogen atoms, as is evident by small spikes in the RDF plot at 0.20 and 0.45 nm (Figure S6a), and (2) TMAO is more likely to interact with the hydrophobic C-terminus than the N-terminus, and it has an even greater affinity than the Gln side chain, despite having a similar structure to the C-terminus (Figure S6b–d). Noteworthily, the order of their charge values in the CHARMM36 force field does not align with the ranking of these RDF values, which can be attributed to steric hindrance that reduces the accessibility of the SP hydrogens to TMAO (i.e., the C-terminus is more solvent-exposed and is more accessible to TMAO) (Table S2); (3) for hydrophobic residues F, M, and L, TMAO also shows affinities at various strength (Figure S6f–h), and (4) TMAO shows a pronounced preference for interacting with charged residues, particularly with Arg and Lys. The surrounding TMAO density around these charged residues is markedly more intense compared to residues that are not charged, which we interpret as evidence that TMAO has a strong affinity for charged residues (Figure S6i,j). Rearrangement of solvent by TMAO around charged residues may reduce charge repulsion between SP monomers and promote dimerization. However, TMAO generally exhibits an exclusion property and is less likely to be found around the protein (Figure S6k). This is further supported by the fact that water solvation is slightly enhanced in the presence of TMAO compared with the solution without TMAO, as the initial RDF peak value increased from 0.75 to 0.78 (Figure S6l). Further RDF calculations were also carried out at 280 (Figure S7) and 360 K (Figure S8). Most interactions showed no significant differences; however, the interaction between TMAO and charged residues did increase slightly as the temperature increased.

**Figure 6:**
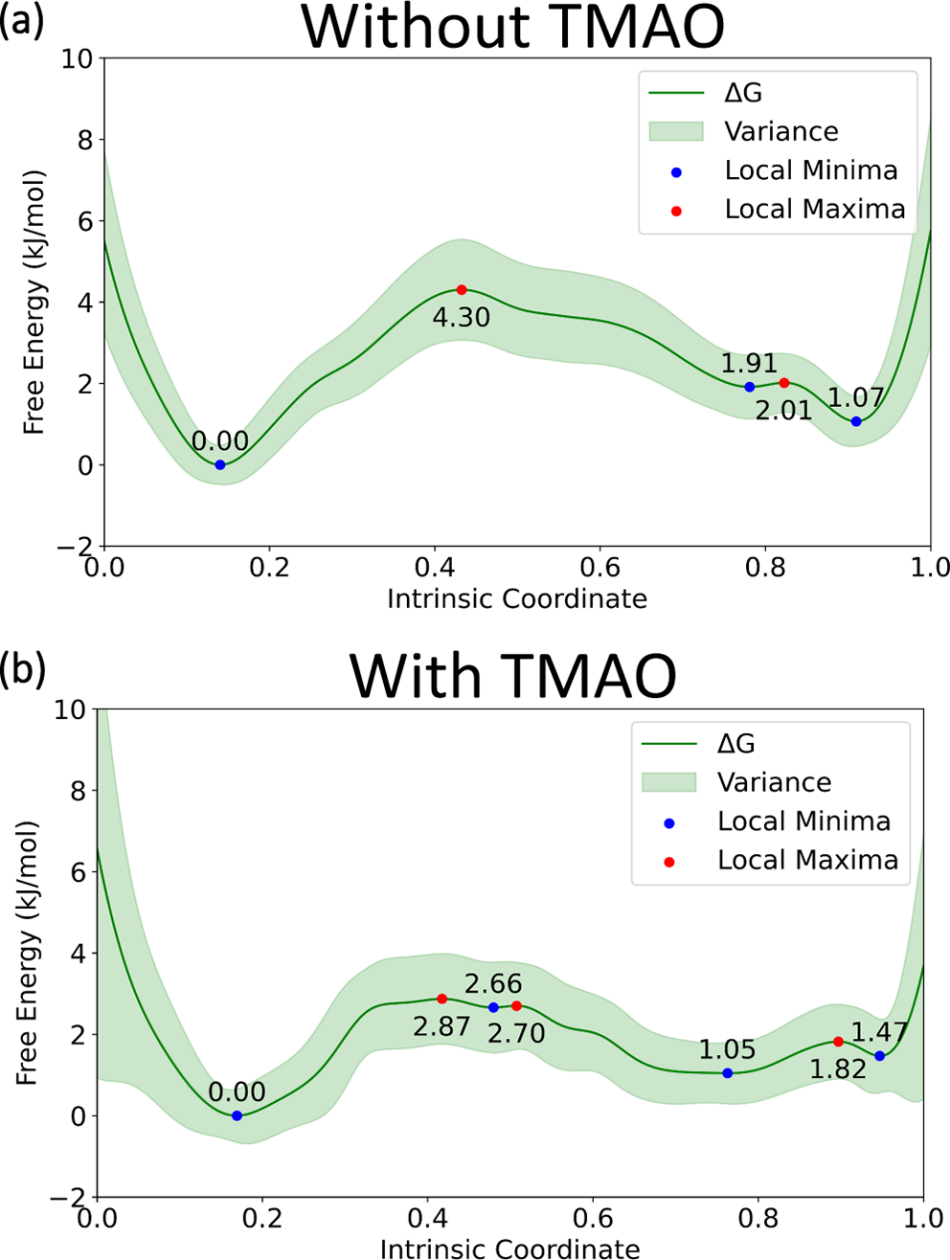
Distance derived free energy profiles (a) without TMAO at *T* = 300 K and (b) with TMAO at *T* = 300 K. Normalized COM distance is selected as the intrinsic coordinate, and smaller intrinsic coordinate values correspond to smaller COM distances. The highlighted band represents variance estimation from bootstrapping.

Since TMAO showed minimal interaction with SP monomers or dimers, we hypothesized that TMAO does not alter dimer formation by direct interaction with SP but rather alters the solvation of the SP molecules. Derived from Max–Boltzmann distributions (Figure S9), estimated free energy profiles (FEP) were constructed for the SP dimerization process (Figure 6). In the FEPs, regions with values lower than 0.3 are recognized as dimers, while values between 0.6 and 1.0 correspond to separated monomers. The transition region is located between these two ranges. The relative energies of dimers and monomers are not altered significantly in either case, which supports our conclusion that TMAO does not bind to SP. We found that the dissociation energy barrier is significantly reduced when TMAO is present in solution, as is evident by the flatter FEP profile (Figure 6a,b). The free energy profiles also provide evidence that TMAO changes the association and dissociation mechanism by making the processes more stepwise, as we observed multiple local minima and local maxima in the transition stage. Herein, we can conclude that TMAO acts as a crowding reagent,^6,35^ entropically changing the FEPs by smoothing the transition from the dimer to monomers. Inspired by the charge reducing phenomenon, further studies should be done with interfacial phase simulations to see how TMAO behaves in a small droplet. Lowering of the free energy barrier for SP dimer formation is consistent with the observation made in the van’t Hoff plots (Figure 3a), which reveals that more dimers are present at lower temperatures when the entropy value is lowered.

### Dimerization of SP Mutants

**Figure 7:**
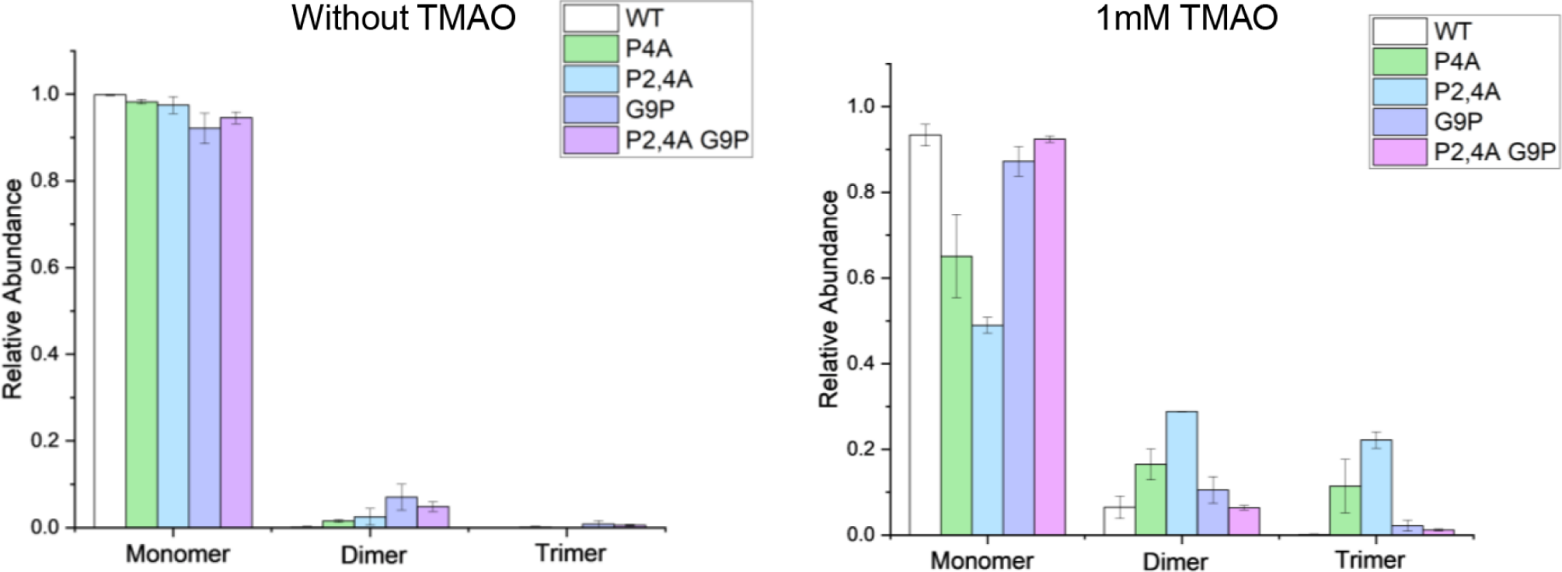
Degree of oligomerization of wt-SP and proline mutants with and without 1 mM TMAO. Values were extracted from MS data (Figure S11) processed using Protein Metrics intact software. Error bars represent standard deviation of replicates (*n* = 3).

Lastly, ambient temperature ESI experiments with 1 mM TMAO were performed on different mutants of SP to provide evidence for the importance of the peptide conformation on SP dimer formation. Our results yield evidence that for many solutions, oligomers of SP increase in abundance when TMAO is inserted into the solution (Figures 7 and S10). Mutants involving P → A (P4A and P2,4A) show a significant increase in oligomer formation compared to wild type SP (wt-SP) (Figures 7 and S11) where P2,4A has the highest degree of oligomerization (Figure S12). We interpret the difference in dimer formation to mean prolines in the *cis* position make the SP structure compact, decreasing dimer interactions. Mutating proline 2 and 4 to *trans*-alanine extends the structure and makes the peptide more susceptible to dimerization. CCS profiles of SP provide evidence that the P4A and P2,4A mutants favor extended conformers compared to wt-SP, which supports our claim (Figure S13). Introduction of proline to the hydrophobic C-terminus (G9P) does not significantly affect oligomerization compared to wt-SP, but significantly decreases the level of oligomer formation of the P2,4A mutant (P2,4A, G9P) (Figures 7 and S11). We interpret the decrease in dimer formation when G9P is introduced as evidence that the *cis* configuration of the proline prevents dimerization of SP, which is supported by the altered CCS profile of the G9P mutant and the P2,4A G9P mutant (Figure S13). These results provide evidence that (1) the removal of *cis*-prolines in the 2 and 4 position significantly increases oligomerization induced by TMAO most likely because dimerization favors the extended conformer^12^ and (2) the inhibition of oligomerization with the G → P mutation indicates that the hydrophobic C-terminus conformation is important in the dimerization process. ESI of SP mutants provides evidence that the conformation of substance P and specifically the *cis*/*trans* configuration of proline residues affect the propensity of SP to dimerize when TMAO is present in the solution.

## Discussion

The experiments performed in this study provide insight into the mechanisms for TMAO stabilization of the protein structure. Cryo-IM-MS data provide evidence that TMAO contributes indirectly to dimerization of the SP molecule, even at a low concentration of TMAO (1 μM). The low concentrations of analytes used in this study draw attention to the unique solution environment of ESI droplets.^36^ The concentration of SP utilized in the experiment was in the low micromolar range. The low concentration of SP is most likely why oligomers with the highest abundance are dimers; however, Figure 7 provides evidence that higher order oligomers are formed when the concentration of TMAO in the solution is increased. Typically, in solution phase studies, the osmolyte concentration used is in the molar range,^27^ but in the MS experiments in this study, micromolar or millimolar concentrations of osmolytes were used to induce observable products of osmolyte interactions. Circular dichroism (CD) studies performed by Rueger et al. show that SP can spontaneously form oligomers in solution via hydrophobic interactions in the mM concentration range,^37^ but the results from cryo-IM-MS show that TMAO enhances oligomer formation in the μM range through an indirect mechanism rather than a direct mechanism. Evidence for an indirect mechanism is the exclusion or absence of TMAO molecules adducted to the dimer complex in the cryo-IM-MS experiment. Experimental data and MD simulations have predicted that protein surfaces exclude TMAO, which may explain why TMAO is not adducted to the [2SP + 4H]^4+^ ions.

Our initial observation of the [2SP + 4H]^4+^ dimer using cryo-IM-MS provided the impetus for follow-up experiments targeted at obtaining thermodynamic data for the TMAO/SP interaction and formation of the SP dimer. Thermodynamic data for the TMAO/SP interaction suggest that TMAO utilizes entropic favorability to interact with the SP molecule at cold temperatures, a process involving reorganization of hydrating water from the peptide backbone to the bulk solution, thereby inducing a solvent-excluded volume effect that preferentially favors hydrophobic interactions (i.e., an indirect mechanism). Exclusion of water from SP by TMAO may allow for increased interactions between SP molecules and an increased probability for dimer formation.

**Scheme 1:**
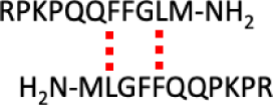
A Representation of a Possible Interface for the Formation of the SP Dimer The interactions involving phenylalanine and leucine residues are similar to those reported by Krone et al. for the dimer, the amyloid-β peptide (16–42).^40^

The thermodynamic data also provide evidence that the entropic favorability of the SP/TMAO complex coincides with a lowering of the entropic barrier for self-assembly. Lower solution temperatures decrease the prevalence of the hydrophobic effect,^38^ whereas in the presence of TMAO, the self-assembly of SP, involving hydrophobic–hydrophobic interactions,^37^ is favored at low temperatures. Hydration of hydrophobic molecules is generally disfavored under ambient conditions owing to an entropic penalty as the hydrating water becomes more ordered; however, reorganizing water molecules around the hydrophobic regions of the molecule is entropically favored at lower solution temperatures.^33,39^ Shea and coworkers showed evidence that small increases in solution temperature (20 K) are sufficient to remove water from hydrophobic regions of biomolecules.^40^ Their results provide a possible explanation of why there is a mechanistic change in the thermodynamics for the dimer formation of SP. As the solution temperature decreases, the solvated state of the hydrophobic region of SP transitions from a “dry” state to a “wet” state. TMAO then displaces water molecules surrounding the hydrophobic (−FFGLM–NH_2_) region of the SP monomers and promotes monomer interaction by lowering the Gibbs free energy barrier for the reaction. The water molecules then form a hydration shell around the hydrophobic region of the dimer complex based on the topography of the hydrophobic surface.^41^ Data from Krone et al. indicate that the interaction between phenylalanine (F) and leucine (L) residues form the interface of the amyloid-β peptide, which were proposed for self-assembly of amyloid-β protofilaments (16–22).^40^ A similar mechanism may contribute to the formation of SP dimers, as the phenylalanine (F) and leucine (L) residues have similar spacing in the SP sequence (Scheme 1), although this is probably not the driving force for dimerization. Moreover, the positioning of the hydrophilic N-terminus (H_3_N^+^–RPKPQQ−) at opposite ends of the dimer serves as hydration centers. At higher solution temperatures, the hydrophobic side chains would become “drier” by reorganizing the water molecules to form a hydration shell around the N-terminal hydrophilic regions of [2SP + 2H]^2+^ ions with concomitant loss of TMAO and dimer formation. This interpretation agrees with the vT-IM-MS data, in which loss in the abundance of [2SP + 2H]^2+^ ions as a function of solution temperature is markedly increased for temperatures greater than ∼293 K.

Our MD simulations provide further indication that TMAO indirectly promotes SP self-assembly. The simulations show that the interaction between TMAO and SP dimer molecules is weak, but the transient interactions form solvent excluded volumes, favoring interactions between SP molecules. FEP data show that the free energy barrier of dimer formation is lowered when TMAO is present, which supports the hypothesis that TMAO indirectly promotes dimer formation. The formation of the dimer itself reduces entropy due to the loss of configurational freedom of the monomers; however, it must be noted that the diversification of transition states increases entropy by providing more possible configurations during the transition from monomer to dimer. Overall, TMAO makes the dimerization process more favorable by stabilizing intermediate states, thereby balancing the entropic and enthalpic contributions to the free energy landscape. HB analysis provides evidence that once the monomers interact, HBs between SP monomers are formed, which further stabilizes the dimer. MD simulation data largely support the conclusions from our experimental data, namely, that TMAO excludes water molecules from SP molecules, which lowers the free energy barrier of SP self-assembly, and that enthalpy in the form of HBs stabilizes the dimer upon self-assembly.

Lastly, results from ESI of the peptide conformation contribute to oligomerization of SP molecules. SP mutants that favor more extended conformations of SP, namely, P4A and P2,4A, result in an increased amount of oligomerization. Most likely, this is a result of the transition from the *cis* configuration of proline to the *trans* configuration of alanine. Conversely, the addition of the G9P mutation reduces the level of oligomerization by introducing the *cis* configuration of proline to the sequence. IM-MS data from Fort et al. provides evidence that differences in CCS from wt-SP are apparent for these mutants, which further supports our claim.^12^ The FEP profiles in Figure 6 suggest that TMAO stabilizes intermediate states, and it is possible that TMAO promotes extended conformations of SP containing these mutations. The difference in oligomer formation provides evidence that conformation affects oligomerization of SP, and it is possible that solution conditions that stabilize extended conformers of SP increase interactions between the SP monomer and thus dimerization propensity.

Further studies should be directed at understanding of the roles by which the nanodroplet environment alters osmolyte–biomolecule interactions. Moreover, the inclusion of experimental data as a complement to MD simulations is important to elucidate the underlying thermodynamic mechanisms (EEC) of osmolyte interactions. The vT-ESI-MS studies reported here provide a new level of specificity that is difficult to achieve in conventional solution phase experiments.

## Conclusions

In summary, the role of TMAO in formation of SP dimers involves a complex interplay. TMAO weakly interacts with SP monomers in the micromolar range and is speculated to exclude water molecules from the surface of the peptide. Thermodynamic data provide evidence that interaction of TMAO with the peptide is dependent on temperature. It is possible that at higher temperatures (>298 K), TMAO interacts with the peptide through HBs, and at low temperatures (<298 K), TMAO interacts with the peptide through hydrophobic interactions. SP dimers are present at high temperatures and low temperatures when TMAO is present in the solution, but they are more abundant at low temperatures when SP transitions from a “dry” state to a “wet” state. Thermodynamic data also provide evidence that the mechanism of dimer formation is different at low temperatures (<298 K) vs high temperatures (<298 K), which correlates with the change in TMAO interaction. MD simulations reveal that TMAO interactions lower the entropic barrier and stabilize intermediate states for SP self-assembly. Once SP molecules interact, HBs form between the monomers and preserve the dimer interaction between SP molecules. Furthermore, ESI of SP proline mutants in solutions containing TMAO shows differences in oligomer abundances, which provides evidence that oligomer formation is altered based on the *cis*/*trans* configuration of prolines at positions 2, 4, and 9. These data showing that TMAO facilitates dimer formation of SP molecules demonstrate that osmolytes can affect protein structure indirectly by altering the environment (i.e., hydration) around them and highlights the importance of discerning the role of enthalpy and entropy in biochemical processes. Moreover, this study provides evidence that *cis*/*trans* configurations of proline residues at positions 2, 4, and 9 alter oligomer formation, and it is possible that TMAO contributes to the stabilization of the conformations that are favorable to self-assembly.

## Supporting Information Available

Additional cryo-IM-MS spectra, MD simulation data, and additional information on oligomerization of SP mutants (PDF)
