# Supplementary material for: Temperature-Dependent Trimethylamine N-Oxide Induced the Formation of Substance P Dimers
Source: J Phys Chem B. 2024 Nov 6;128(46):11369–78. doi: 10.1021/acs.jpcb.4c04951 (PMC11586895; doi:10.1021/acs.jpcb.4c04951)
Supplement: Supplementary file 1 — jp4c04951_si_001.pdf [file jp4c04951_si_001.pdf]

**Supporting Information**

# Temperature-Dependent Trimethylamine N-Oxide Induced the Formation of Substance P Dimers

Carter Lantz, Zhenyu Xi, Robert L. Rider, Thomas E. Walker, Michael Hebert and David H. Russell*

Department of Chemistry
Texas A&M University
College Station, TX 77843

*Corresponding author: russell@chem.tamu.edu

**Table of Contents** **Page**

| Table of Contents | Page |
|---|---|
| Figure S1 | S2 |
| Figure S2 | S2 |
| Figure S3 | S3 |
| Figure S4 | S4 |
| Figure S5 | S5 |
| Figure S6 | S6 |
| Figure S7 | S7 |
| Figure S8 | S8 |
| Figure S9 | S9 |
| Figure S10 | S9 |
| Figure S11 | S10 |
| Figure S12 | S11 |
| Figure S13 | S12 |
| Table S1 | S13 |
| Table S2 | S13 |
| References | S13 |

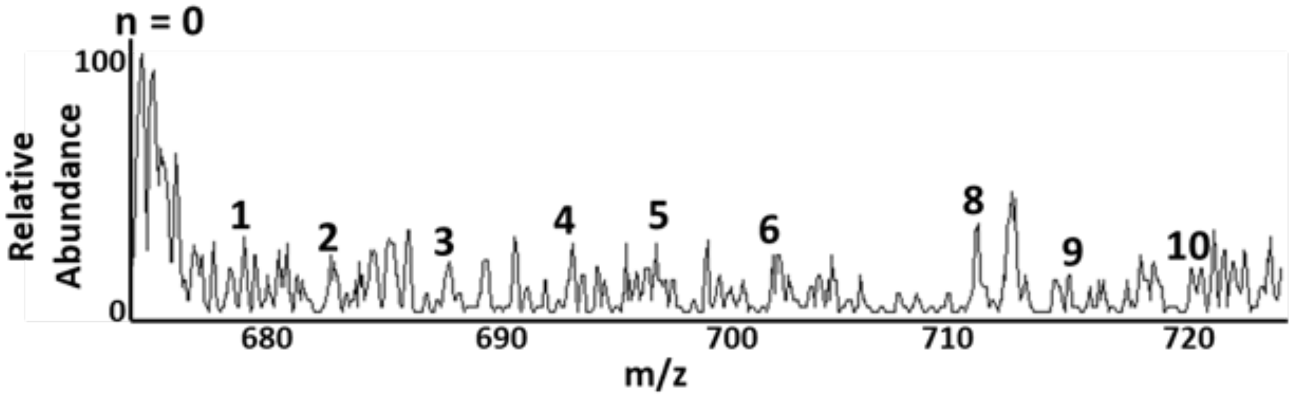


**Figure S1.** A cryo-ion mobility mass spectrum of an SP solution revealing signals corresponding to the SP dimer and the SP dimer with up to 10 water molecules bound. Each water molecule binding event is spaced by 4.5 *m/z*.

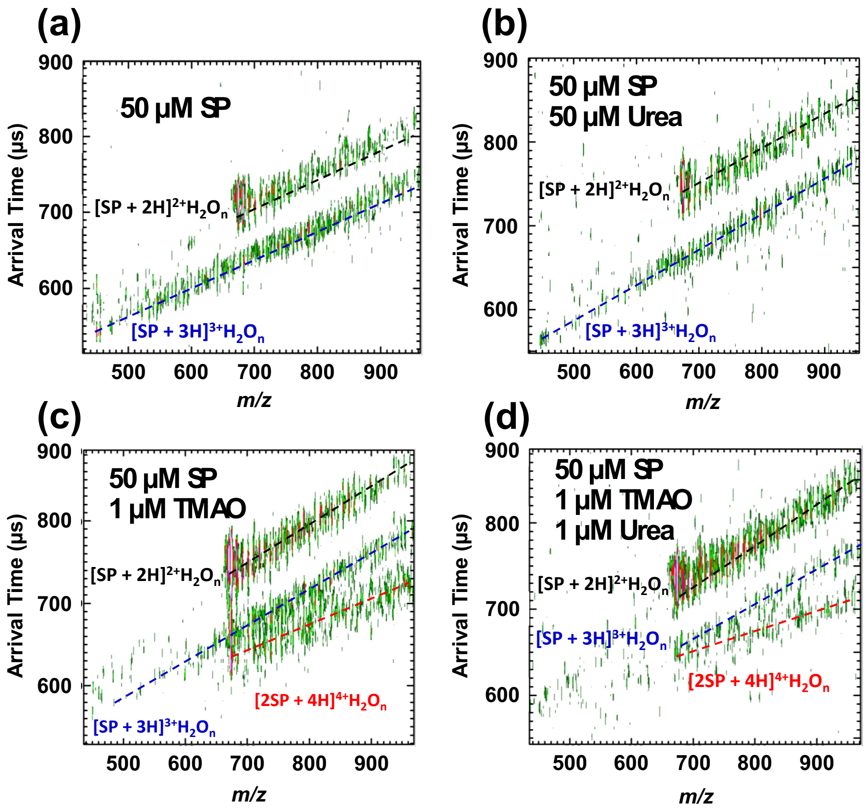


**Figure S2.** Plots of ion mobility arrival-time vs. *m/z* for hydrated SP hydrated cluster ions obtained from solutions of **(a)** 50 µM SP, **(b)** 50 µM SP with 50 µM urea, **(c)** 50 µM SP with 1 µM TMAO, and **(d)** 50 µM SP, 1 µM TMAO, and 1 µM urea. The colors correspond to the abundance of the signals in the spectra. Note the appearance and disappearance of the SP dimer when comparing the various experiments with and without TMAO.

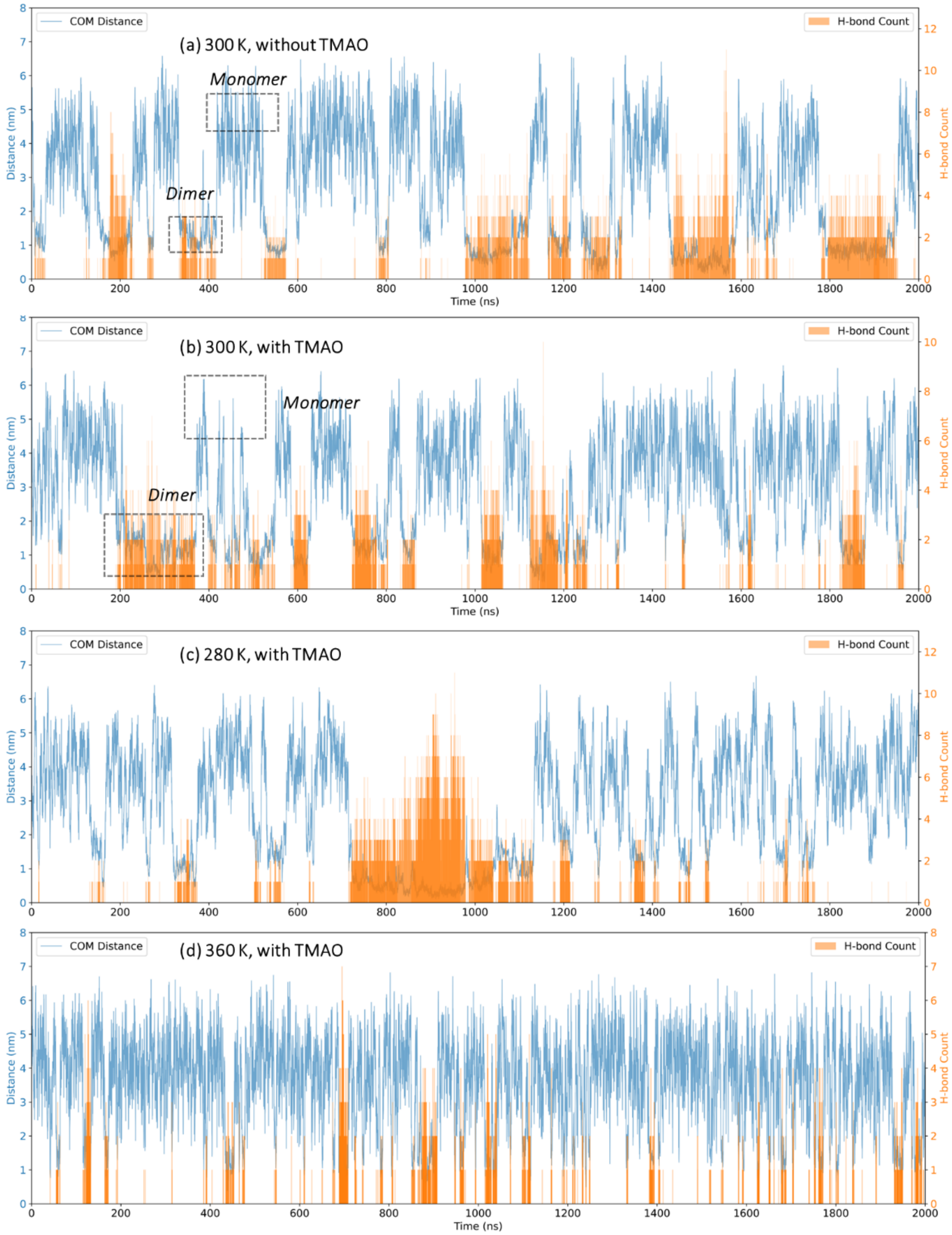


**Figure S3.** Distance between monomeric SP center of mass and SP-SP HB counts, (a) without TMAO at 300 K, (b) with TMAO at 300 K, (c) with TMAO at 280 K, (d) with TMAO at 360 K.

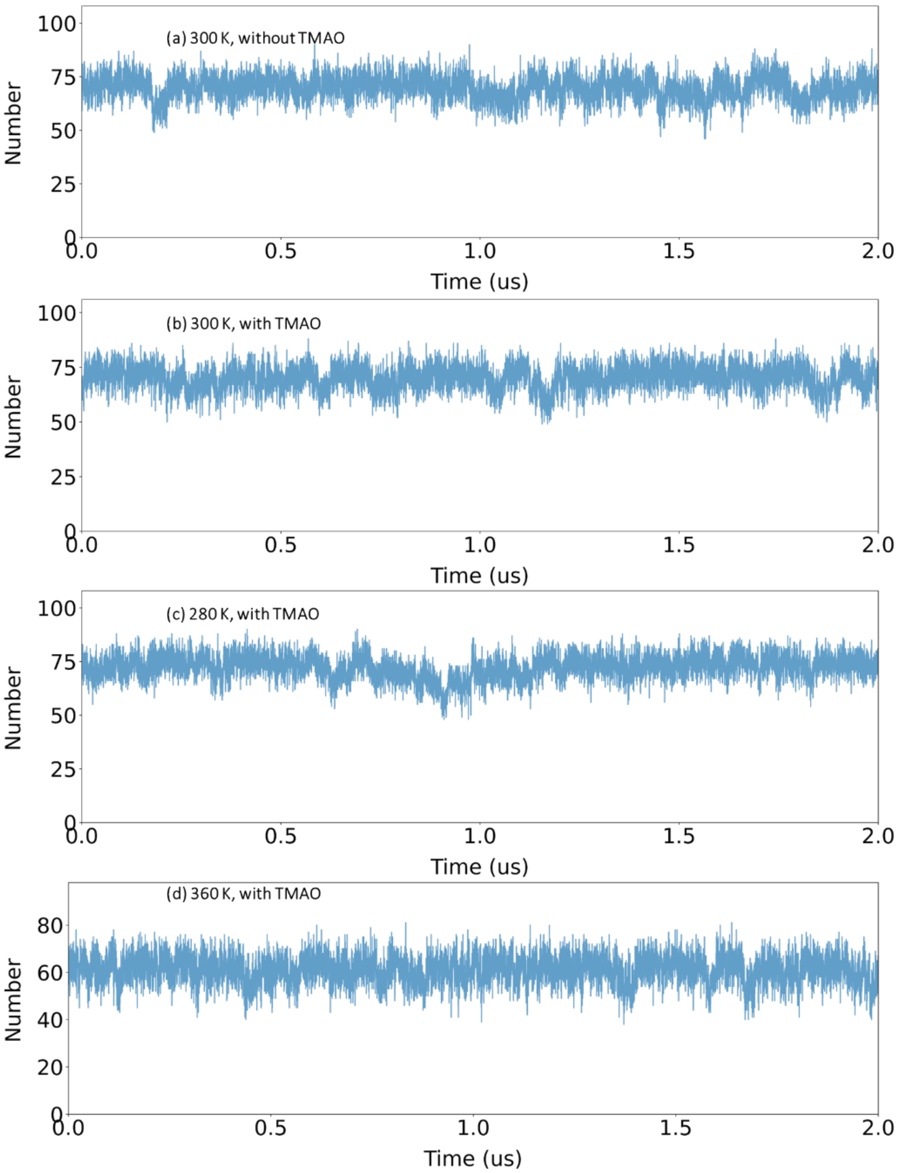


**Figure S4.** SP-water HB counts as a function of time, (a) without TMAO at 300 K, (b) with TMAO at 300 K, (c) with TMAO at 280 K, (d) with TMAO at 360 K.

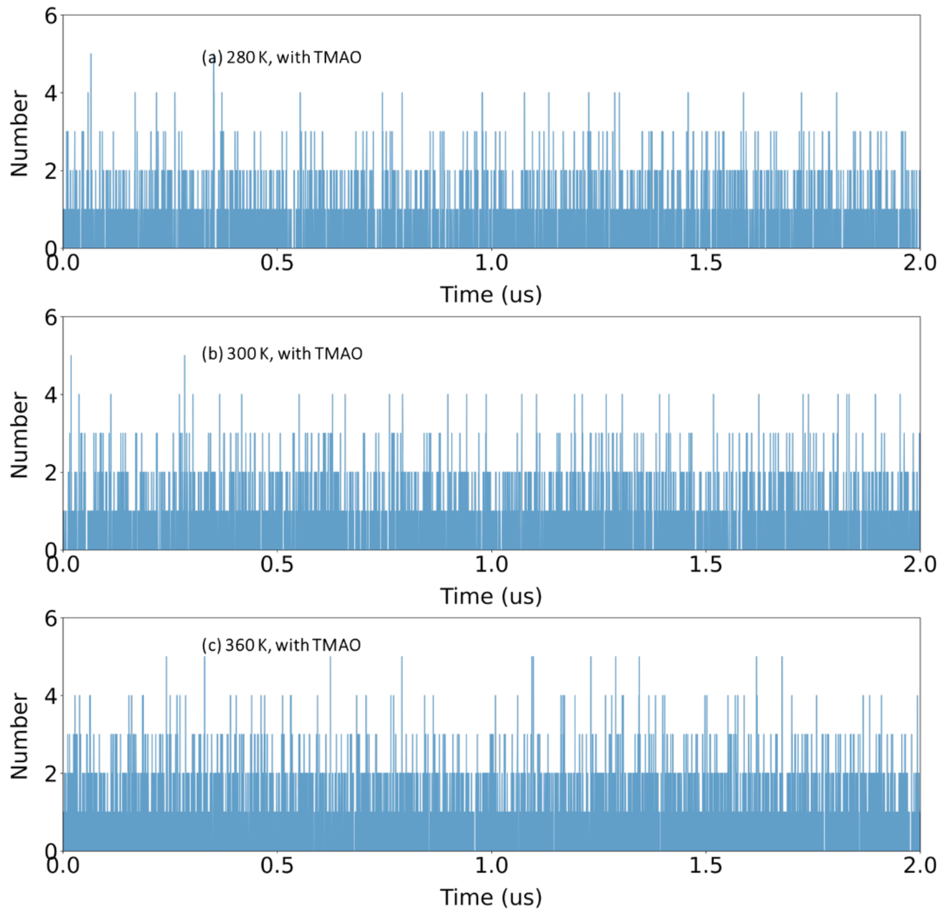


**Figure S5.** SP-TMAO HB counts as a function of time, (a) with TMAO at 280 K, (b) with TMAO at 300 K, and (c) with TMAO at 360 K.

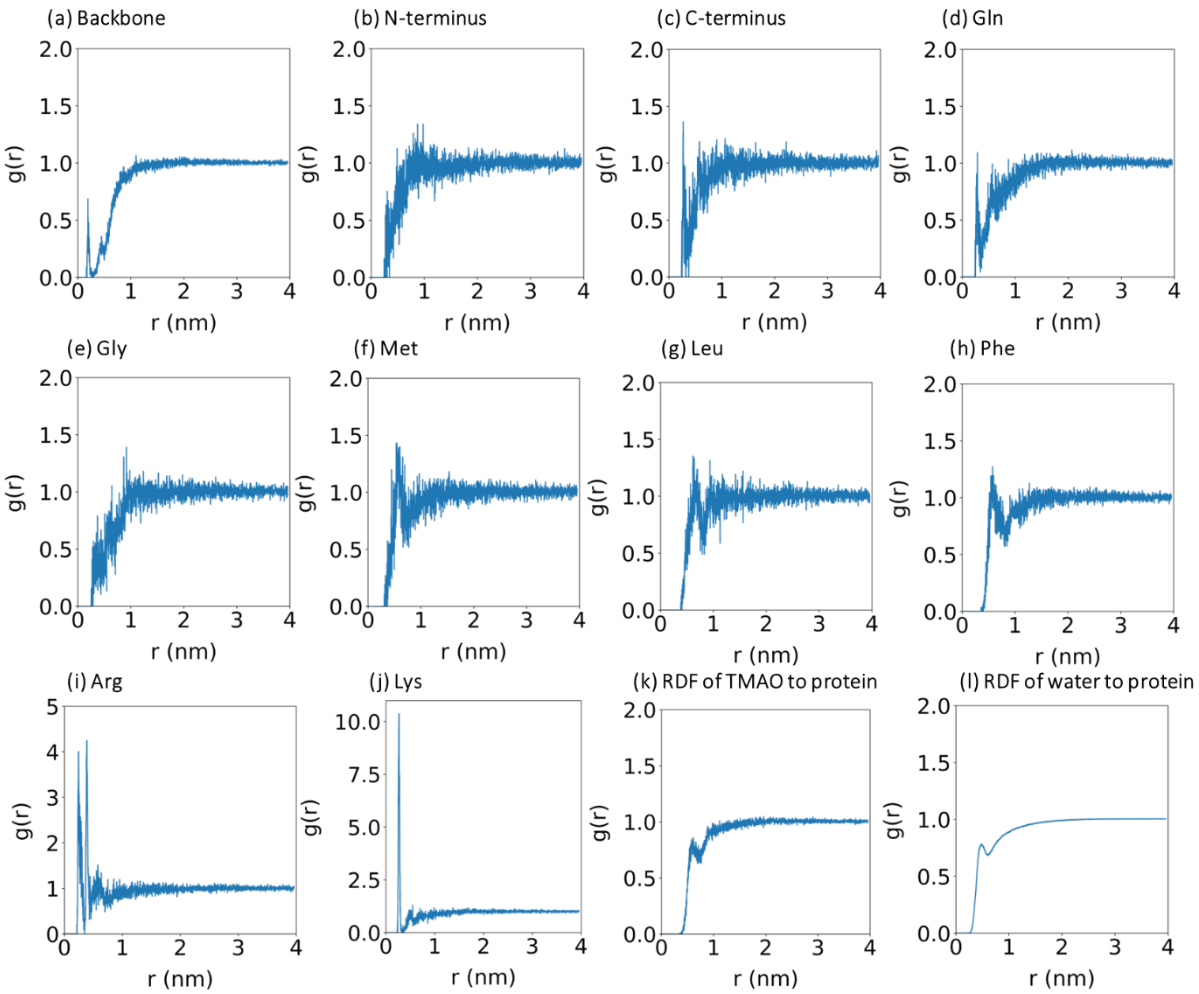


**Figure S6.** Radial distribution functions (RDF) for TMAO at 300 K interacting with the **(a)** backbone, **(b)** N-terminus, **(c)** C-terminus, **(d)** glutamine residue, **(e)** glycine residue, **(f)** methionine residue, **(g)** leucine residue, **(h)** phenylalanine residue, **(i)** arginine residue, and **(j)** lysine residue on the SP molecule. RDF plots revealing the interaction of **(k)** TMAO with the entire SP molecule and **(l)** SP with water.

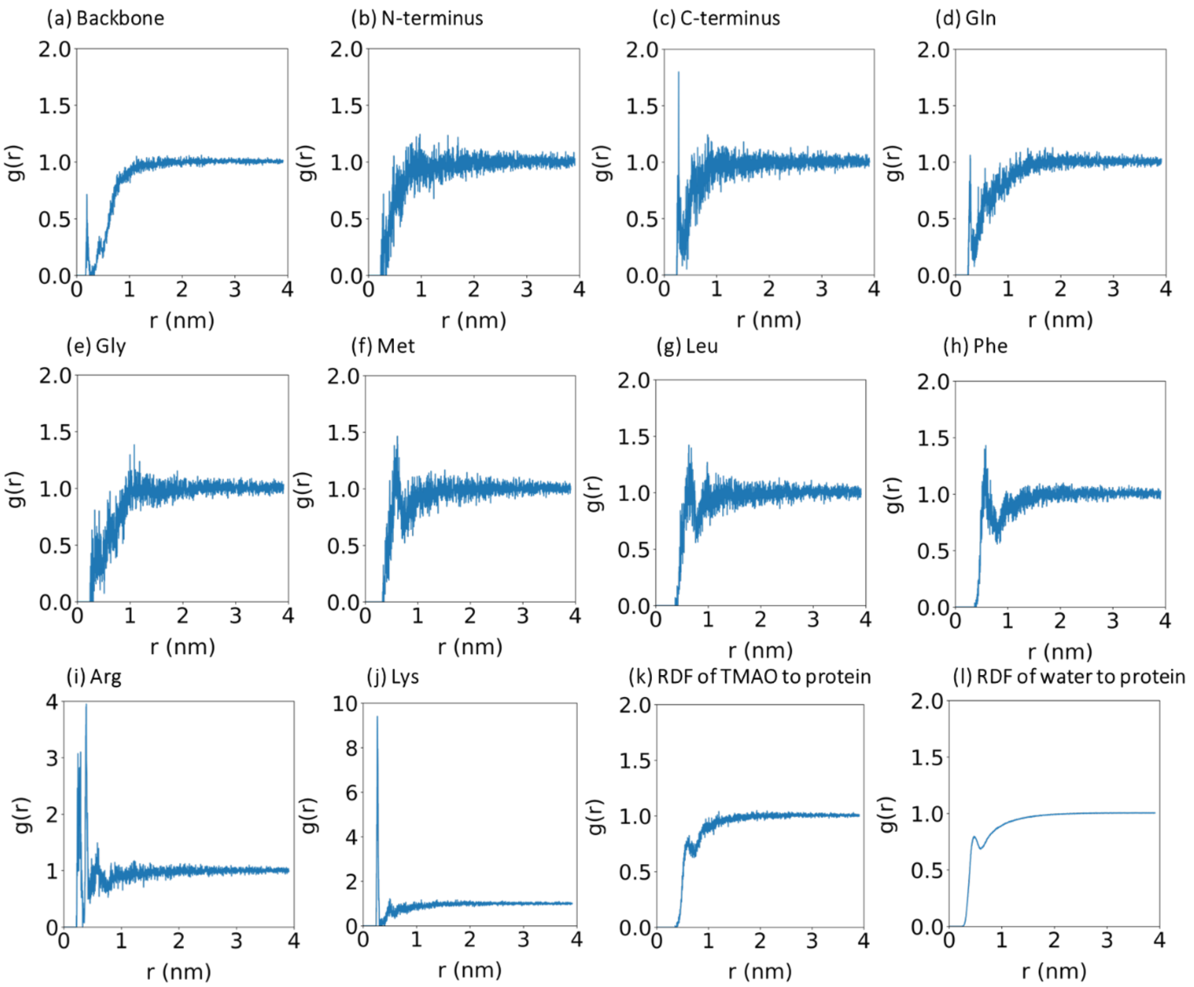


**Figure S7.** Radial distribution functions (RDF) for TMAO at 280 K interacting with the **(a)** backbone, **(b)** N-terminus, **(c)** C-terminus, **(d)** glutamine residue, **(e)** glycine residue, **(f)** methionine residue, **(g)** leucine residue, **(h)** phenylalanine residue, **(i)** arginine residue, and **(j)** lysine residue on the SP molecule. RDF plots revealing the interaction of **(k)** TMAO with the entire SP molecule and **(l)** SP with water.

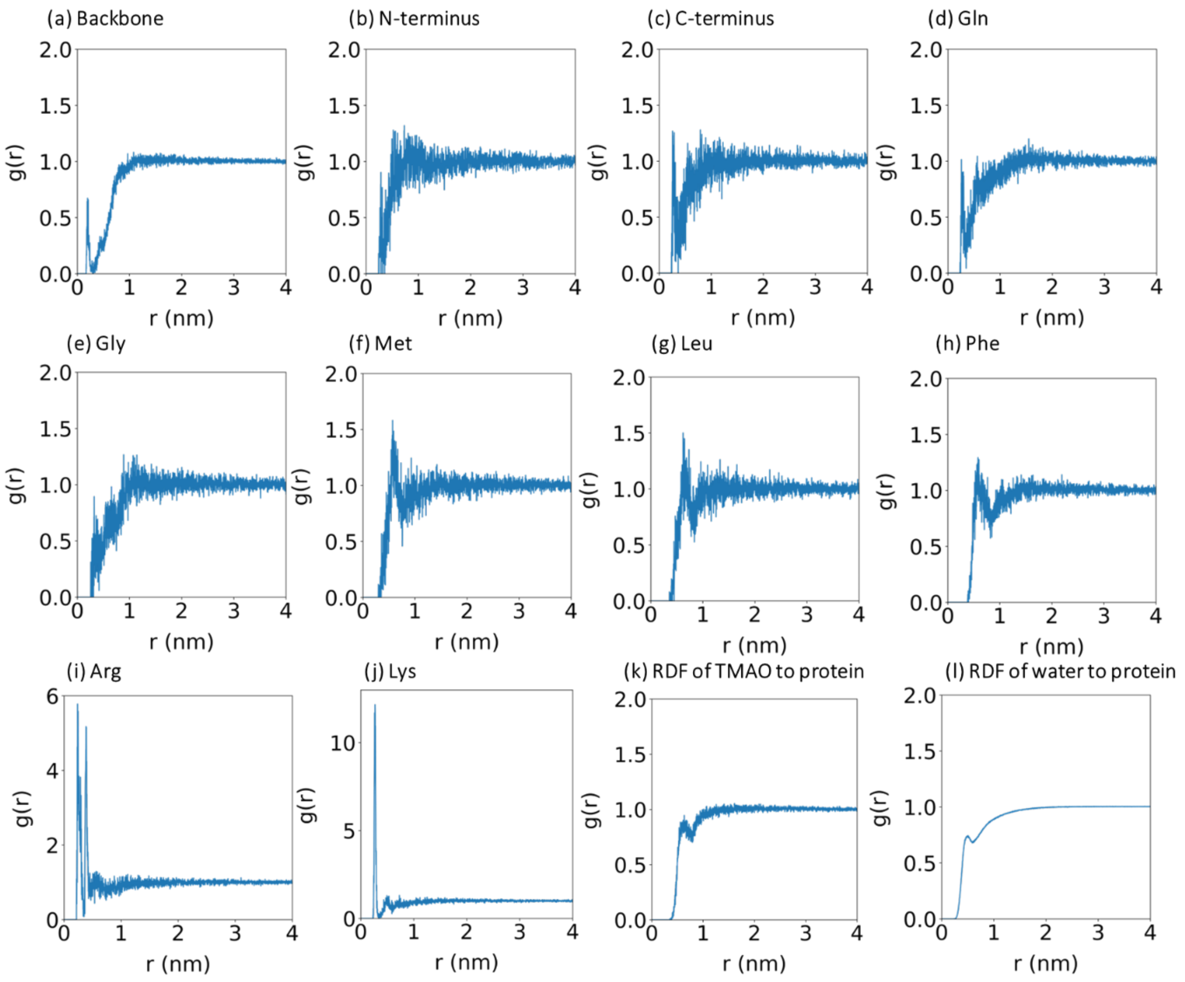


**Figure S8.** Radial distribution functions (RDF) for TMAO at 360 K interacting with the **(a)** backbone, **(b)** N-terminus, **(c)** C-terminus, **(d)** glutamine residue, **(e)** glycine residue, **(f)** methionine residue, **(g)** leucine residue, **(h)** phenylalanine residue, **(i)** arginine residue, and **(j)** lysine residue on the SP molecule. RDF plots revealing the interaction of **(k)** TMAO with the entire SP molecule and **(l)** SP with water.

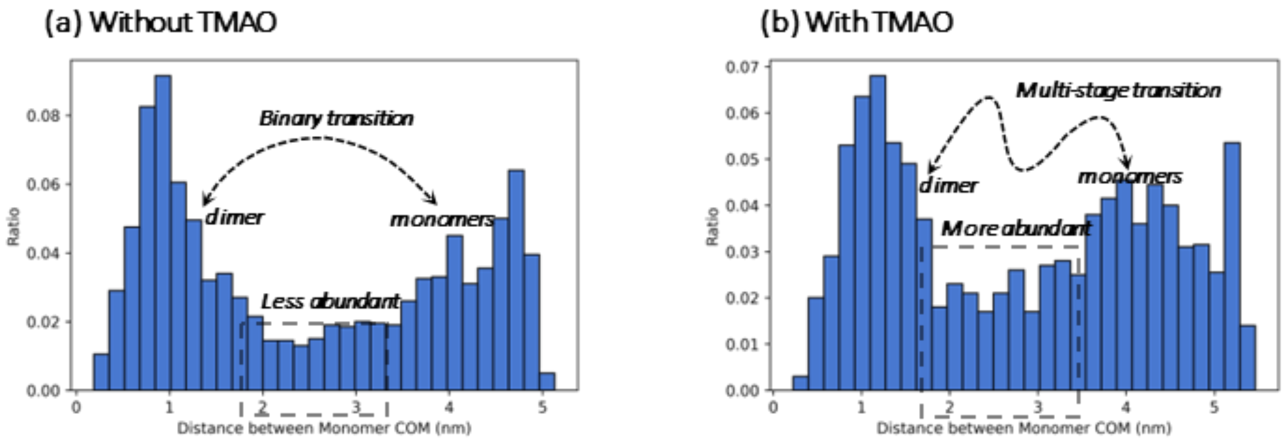


**Figure S9.** Center of mass distributions between two SP molecules **(a)** without TMAO and **(b)** with TMAO. The increase in ratio between 2 nm and 3 nm (dashed box) aligns with a decreased energy barrier for dimerization when TMAO is present.

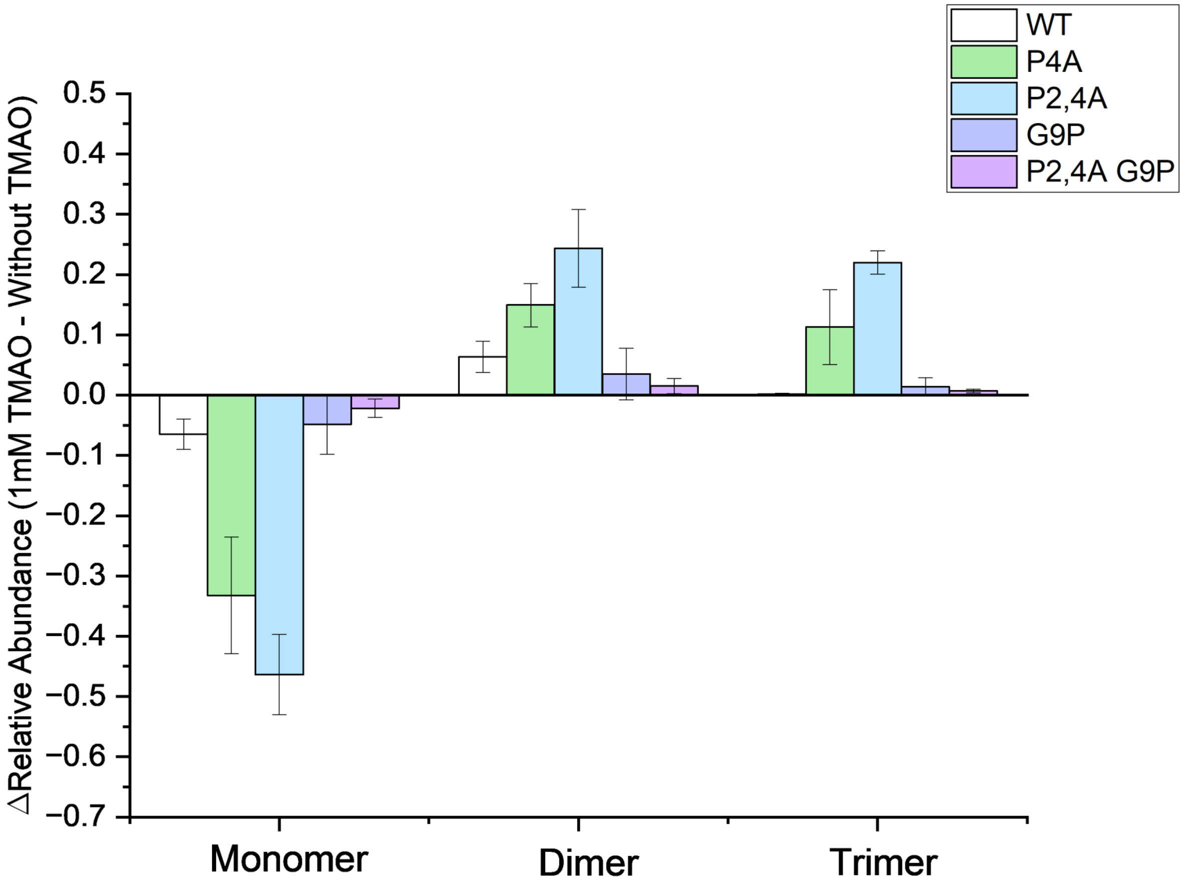


**Figure S10:** Difference of oligomer relative abundance between mutants in the presence of 1 mM TMAO and samples without TMAO.

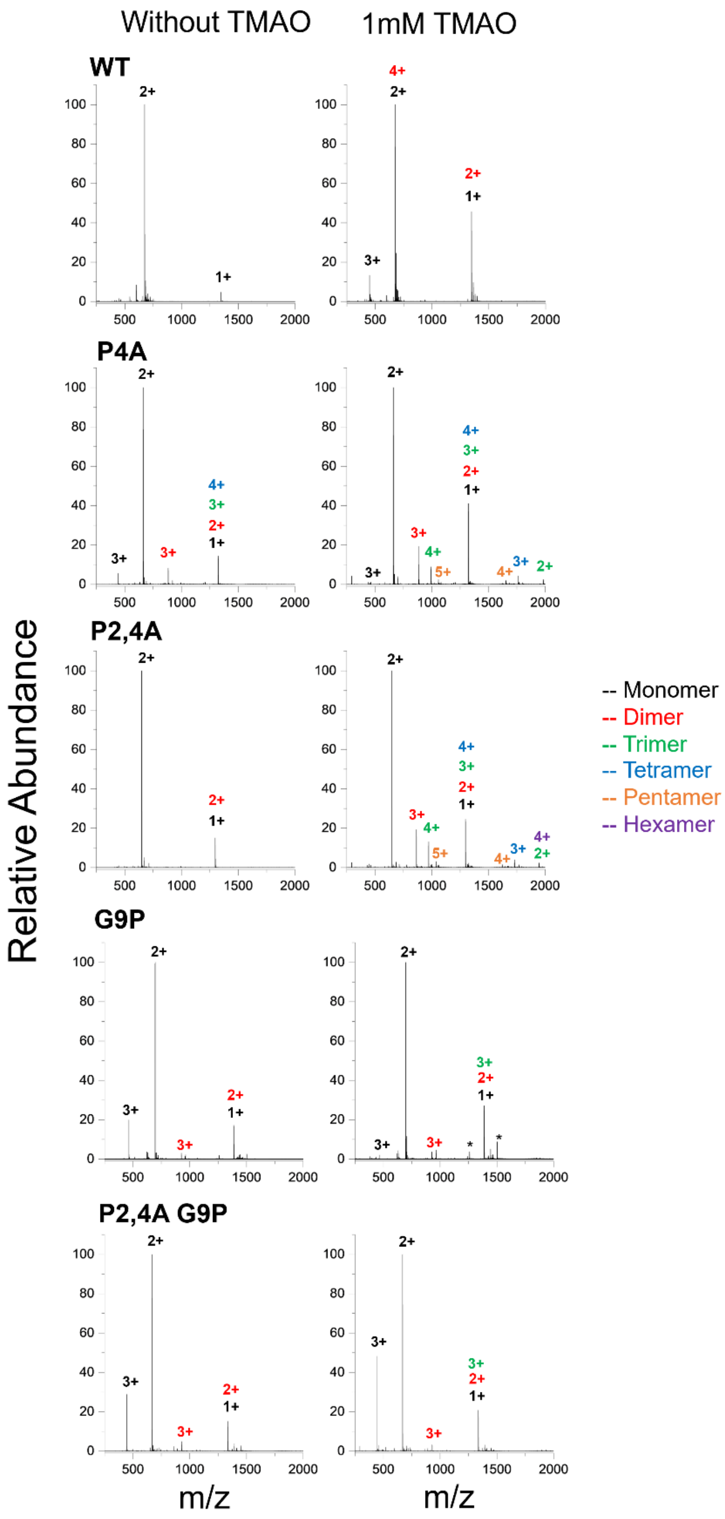


**Figure S11**: Electrospray ionization mass spectra of WT-SP and mutant-SP with and without TMAO. Labels indicate the identity and charge state of the oligomers present in the spectrum.

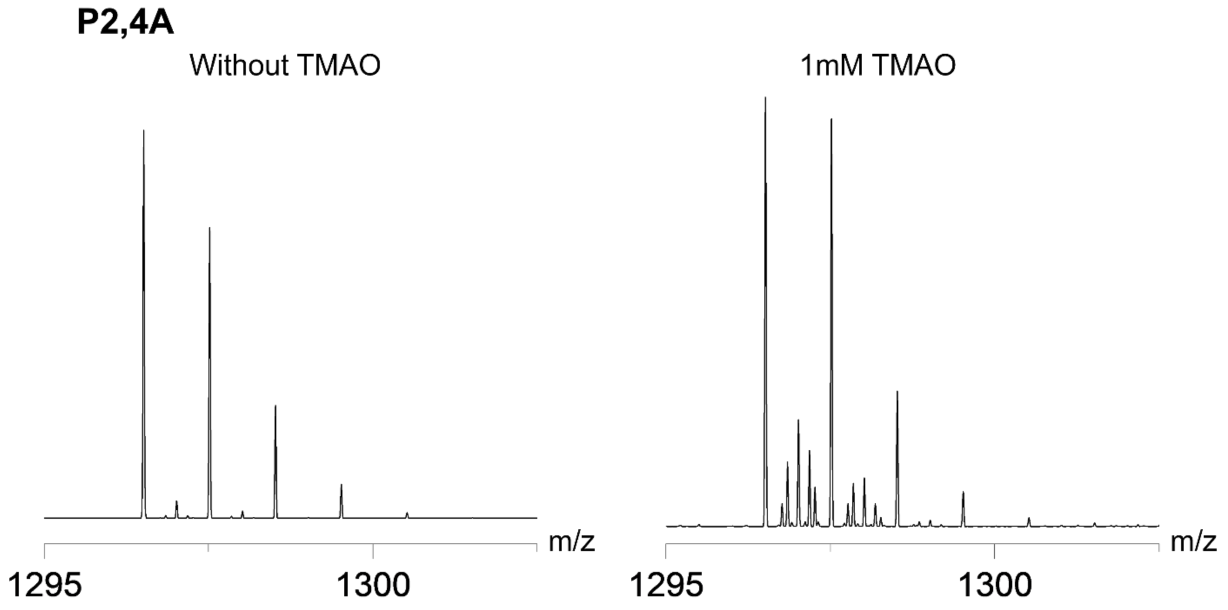


**Figure S12**: A zoomed in mass spectrum of the region with signals corresponding to the 1+ monomer charge state of P2,4A with and without 1 mM TMAO. The peaks in between the monomer isotopes correspond to oligomers present in the solution. The mass spectra collected when TMAO is present in the solution reveal the presence of numerous oligomer states up to a tetramer.

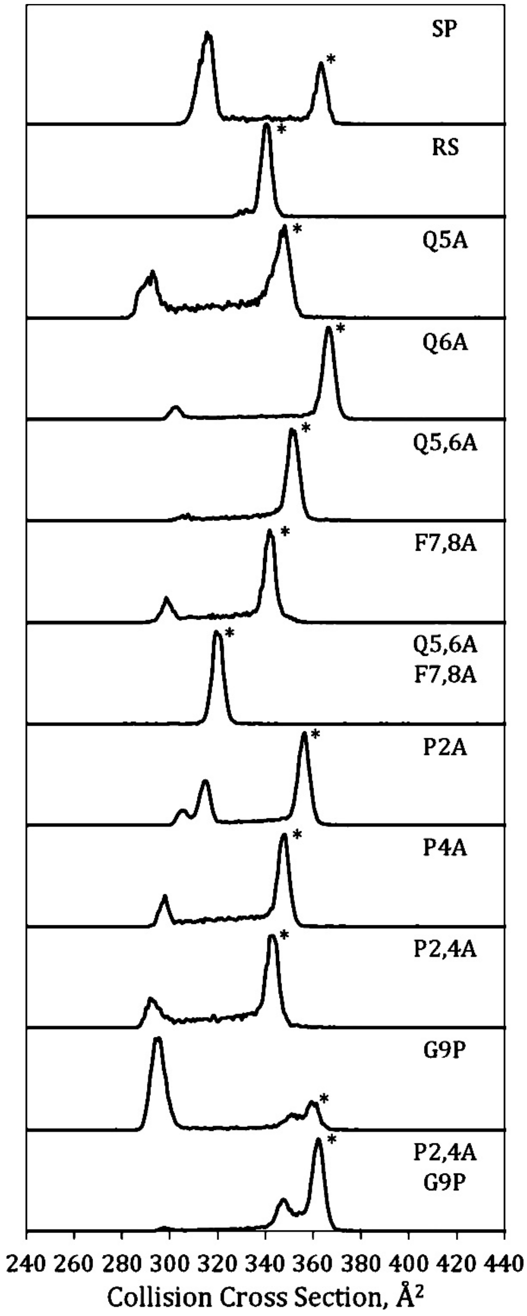


**Figure S13:** CCS profiles of SP $[M + 3H]^{3+}$ ions and intrinsic size-corrected CCS profiles of SP mutant $[M + 3H]^{3+}$ ions. Shifts in the CCS profiles between SP and SP mutant ions arise from variations in ion packing efficiency that are not represented by the intrinsic size parameter shift. An asterisk denotes the elongated B conformer of each peptide ion. Assignment of the elongated conformer for peptide ions that have a single peak distribution was determined through collisional activation studies. Reprinted from Fort et al. J. Phys. Chem. B 2014, 118, 14336-44.[1]

**Table S1.** Thermodynamic values for SP dimer formation and TMAO Adduct formation.

| | Mechanism | ΔH | -TΔS | ΔG | Notes |
|---|---|---|---|---|---|
| Dimer Formation | Low T | -26.4 | 8.3 | -18.1 | T = 278 K |
| | | -26.4 | 8.8 | -17.6 | T = 293 K |
| | High T | -71.1 | 53.6 | -17.5 | T = 293 K |
| | | -71.1 | 64.6 | -6.6 | T = 353 K |
| TMAO Adduct Formation | Low T | 3.7 | -17.7 | -14.0 | T = 278 K |
| | | 3.7 | -19.0 | -15.3 | T = 298 K |
| | High T | -22.2 | 6.7 | -15.5 | T = 298 K |
| | | -22.2 | 7.5 | -14.7 | T = 333 K |

**Table S2.** Proton type, with charge value and ordered peak value.

| Proton type | Charge value | g(r) peak value |
|---|---|---|
| BB | 0.31 | 0.53 |
| Gln | 0.30/0.32 | 0.94 |
| NT | 0.34 | 1.21 |
| CT | 0.30/0.32 | 1.50 |
| Arg | 0.46 | 4.28 |
| Lys | 0.33 | 9.67 |

**References**


1. Fort, K. L.; Silveira, J. A.; Pierson, N. A.; Servage, K. A.; Clemmer, D. E.; Russell, D. H., From Solution to the Gas Phase: Factors That Influence Kinetic Trapping of Substance P in the Gas Phase. *The Journal of Physical Chemistry B* **2014,** *118* (49), 14336-14344.
